# Automated Quantification of Photoreceptor alteration in macular disease using Optical Coherence Tomography and Deep Learning

**DOI:** 10.1038/s41598-020-62329-9

**Published:** 2020-03-27

**Authors:** José Ignacio Orlando, Bianca S. Gerendas, Sophie Riedl, Christoph Grechenig, Anna Breger, Martin Ehler, Sebastian M. Waldstein, Hrvoje Bogunović, Ursula Schmidt-Erfurth

**Affiliations:** 1 Department of Ophthalmology, Medical University of Vienna, Waehringer Guertel 18-20, 1090 Vienna, Austria; 2 Department of Mathematics, University of Vienna, Vienna, 1090 Austria

**Keywords:** High-throughput screening, Image processing, Machine learning, Prognostic markers, Computer science

## Abstract

Diabetic macular edema (DME) and retina vein occlusion (RVO) are macular diseases in which central photoreceptors are affected due to pathological accumulation of fluid. Optical coherence tomography allows to visually assess and evaluate photoreceptor integrity, whose alteration has been observed as an important biomarker of both diseases. However, the manual quantification of this layered structure is challenging, tedious and time-consuming. In this paper we introduce a deep learning approach for automatically segmenting and characterising photoreceptor alteration. The photoreceptor layer is segmented using an ensemble of four different convolutional neural networks. En-face representations of the layer thickness are produced to characterize the photoreceptors. The pixel-wise standard deviation of the score maps produced by the individual models is also taken to indicate areas of photoreceptor abnormality or ambiguous results. Experimental results showed that our ensemble is able to produce results in pair with a human expert, outperforming each of its constitutive models. No statistically significant differences were observed between mean thickness estimates obtained from automated and manually generated annotations. Therefore, our model is able to reliable quantify photoreceptors, which can be used to improve prognosis and managment of macular diseases.

## Introduction

Diabetic macular edema (DME) and retinal vein occlusion (RVO) are the two most frequent causes of visual impairment in the working age population^1,2^. In 2017, 425 million individuals were suffering from diabetes worldwide and about 10% of these developed vision-threatening DME^1^. This number is estimated to increase to 629 million people worldwide by 2045^3^. With RVO, 14–19 million adults are affected globally^2^. Both conditions are characterised by pathological accumulation of fluid in the sensitive macular layers, severely affecting the central photoreceptors, either causing disruptions in layer continuity^4–6^ or abnormal changes in thickness, including irreversible photoreceptor loss^7^. Functional examinations with microperimetry in other macular diseases have highlighted the topographical association of photoreceptor loss with focal vision impairment^8^. Particularly when this loss is seen foveally, visual impairment is often irreversible and can significantly affect quality of life^8^.

**Figure 1:**
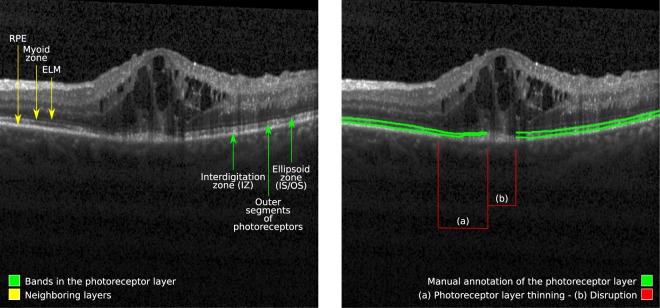
Photoreceptors of a patient with retinal vein occlusion, as observed through the central B-scan of a fovea-centred OCT scan. Left: bands comprising the photoreceptors (green) and neighbouring layers (yellow). Right: manual annotation of the photoreceptor layer as performed by a retina expert (green). (**a**) Thinning in the photoreceptor layer. (**b**) Disruption due to photoreceptor cell death.

Optical coherence tomography (OCT) is the state of the art imaging modality for assessing and evaluating retinal changes associated with DME and RVO. OCT scans can be used to quantify relevant biomarkers such as thickness of the central retinal layers^9^ or the amount of intraretinal cystoid and/or subretinal fluid^10^. In addition, OCT allows to visually assess the photoreceptor layer, which appears as a multi-layered structure with hyperreflective and hyporreflective bands located between the outer limit of the myoid zone (or the inner limit of the ellipsoid zone, or IS/OS) and the inner interface of the retinal pigment epithelium (RPE) (Fig. 1)^11^. Damage to the integrity of the photoreceptors and abnormal changes in their thickness have been previously linked to visual acuity loss in RVO^7^, while intravitreal ranibizumab injections have been observed to facilitate restoration of photoreceptors in DME^12^. In general, alteration of photoreceptor integrity has been identified as the most important imaging biomarker for prediction of visual acuity in DME^13^. Hence, reliably and precisely quantifying the condition of the photoreceptors is essential to improve our understanding of macular function in diagnosis and therapy.

Such a challenging task can be addressed by the incorporation of computerised models based on artificial intelligence and machine learning^14,15^, which have demonstrated to be useful in numerous applications in OCT^16^. These methods can reduce the burden of the tedious and time consuming manual delineation of the photoreceptors by automatically providing accurate segmentations and allowing to quantify alteration/loss of photoreceptors in large populations as well as individual patients.

Selecting the optimal machine learning model for solving the problem is essential, as certain designs might favour some solutions over others. This behaviour is similar to what we expect from different human observers: depending on their knowledge, expertise and visual skills, they will consider specific characteristics of the structures and decide where the edges of the region of interest are located. A natural way to overcome this issue would be to leverage their diverse opinion and produce a consensus annotation based on a voting strategy by averaging. However, in a clinical setting this is costly—as it requires to work with multiple experts—and extremely time consuming.

In this paper we introduce a novel deep learning-based algorithm for automatically segmenting the photoreceptor layers in routine OCT scans. Inspired by the idea of leveraging the opinions of different experts, we propose to train and integrate a series of complementary U-shaped convolutional neural networks architectures. Our ensemble is able to produce not only accurate segmentations, but also consistent photoreceptor thickness maps and disagreement maps that can be used by human experts to identify areas with pathological photoreceptors or errors in the segmentation.

## Materials and Methods

### Study population and OCT images

40 OCT datasets of 40 patients (16 DME, 24 RVO) were acquired by reading centre-certified masked operators at multiple clinical sites, and raw data was uploaded to the Vienna Reader centre (VRC, Department of Ophthalmology and Optometry, Medical University of Vienna, Austria) for subsequent analysis. All scans were captured using a Spectralis OCT device (Heidelberg Engineering, Heidelberg, Germany) with the same predefined image protocol, providing volumetric scans composed of 49 B-scans, each containing 512 A-scans, covering an approximate retinal area of 6 × 6 mm. The images were randomly selected before starting the manual annotation process from different clinical studies and from different disease timepoints, to ensure no bias in data selection and enough variability in disease appeareance. Volumes with low quality B-scans were not included as those B-scans were considered non-diagnostic.

All patients gave informed consent prior inclusion in the respective multi-centre clinical trials and in- and exclusion criteria were the same for all the patients of one. Both the respective prospective studies as well as this post-hoc analysis adhered to the tenets of the Declaration of Helsinki and the standards of Good Scientific Practice of the Medical University of Vienna. The presented study was approved by the Ethics Committee of the Medical University of Vienna, Vienna, Austria (1246/2016).

### Manual annotation protocol and data organization

The automated approach presented in this study is a supervised learning algorithm, which implies that it requires manually annotated images for training. Additionally, ground truth segmentations are needed to compare the outputs of the system with respect to a human expert outcome.

The Iowa Reference Algorithm (Retinal Image Analysis laboratory, Iowa Institute for Biomedical Imaging, Iowa City, IA, USA)^17^ was applied on each B-scan in our 40 OCT scans database to identify the IS/OS and the OB-OPR interfaces. Subsequently, a group of trained readers manually corrected the delineation curves using an in-house software tool, capturing the area between the top of the IS/OS junction and the outer boundary of the third hyperreflective outer retinal band (OPR/COST/IZ). A senior retina specialist supervised the segmentation process to ensure proper labelling of the region of interest, and corrected the resulting masks when necessary. Whenever this retina expert was unsure about the correct annotation of the region, she consulted up to three additional retinal specialists for independent or group discussion, and a consensus annotation was achieved. In case of low quality A-scans, the experts interpolated their annotation from the neighboring B-scans and A-scans, relying on their knowledge of the particular disease course of the patient.

The 40 OCT datasets were randomly divided into a training, validation and test set, each of them comprising 25, 3 and 12 volumes, respectively. The training set was used for learning all the constitutive models of our ensemble, and the validation set was applied for model selection (e.g. parameter calibration and neural network design). The test set was not used until the final evaluation to ensure a proper estimation of the generalisation ability of our approach. In all the cases, a similar proportion of RVO and DME cases was preserved to avoid any disease-based bias.

In order to estimate the inter-grader variability, a retina expert (different from the four experts mentioned above) manually annotated a subsample of B-scans randomly selected from the test set. The same protocol used for generating the gold standard labelling was applied, but without any consensus grading and discussion. In particular, three random samples of 2 B-scans were extracted per each OCT volume, each sample located within the regions of an early treatment diabetic retinopathy study (ETDRS) grid at minimum/maximum distance of 0/1, 1/3 and 3/6 mm from the fovea. Such a sampling strategy allows to compare the automated approach and the human observer’s discretion in regions that are affected by the disease at variable levels, without requiring the expert to fully annotate all the 12 volumes.

All graders have been trained at one of the largest European retina departments with the largest European reading center for standardized retinal image analysis (Vienna Reading Center). All ophthalmologist from the research group have finalized or will finalize their clinical rotations as retina specialists and have 2–10 years of experience in retinal image analysis besides their regular ophthalmology training.

### Segmentation approach

Our segmentation approach is a deep learning method based on an ensemble of U-shaped fully convolutional neural networks (FCNNs). A conceptual representation of these networks is included in the Supplementary Material Fig. 1.

**Figure 2:**
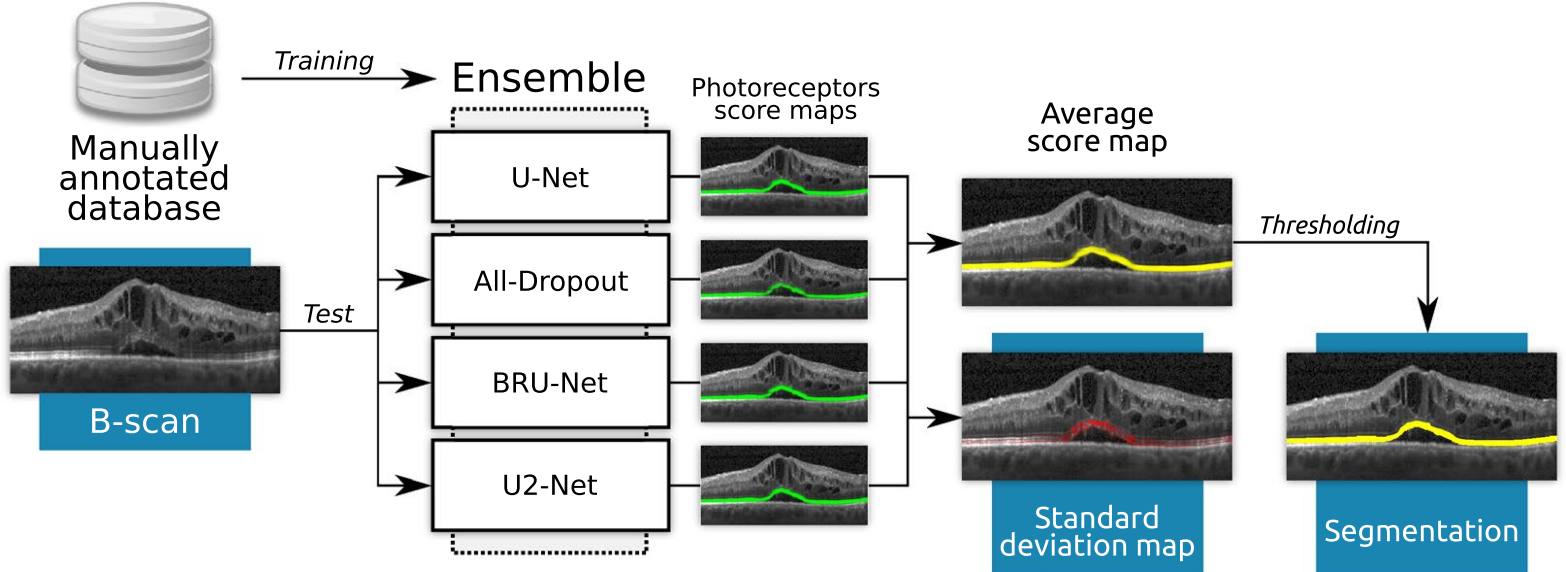
A schematic representation of our automated method for photoreceptor layer segmentation based on an ensemble of U-shaped fully convolutional neural networks. Four different architectures were trained from a database of manually annotated B-scans. Given an unlabelled B-scan, the output score maps of the neural networks are averaged to retrieve a mean score map, and their pixel-wise standard deviation is computed to produce an uncertainty map. The score map is finally thresholded to retrieve a binary representation of the photoreceptors.

  Figure 2 presents a schematic representation of the proposed algorithm. For a formal definition of an ensemble, the interested reader could refer to Supplementary Material Section 1. Given a set of manually annotated B-scans, we trained four different U-shaped FCNN models: three of them are inspired in the U-Net^18^, the BRU-Net^19^ and the All-Dropout^20^ architectures, while the fourth one corresponds to our U2-Net^21^. The U-Net was selected due to its standard design, whereas the BRU-Net was chosen based on its performance for retinal layer segmentation in pathological OCT scans. On the other hand, the All-Dropout architecture was selected due to its improved generalisation ability, which we hypothesised might help to better deal with healthy areas. These architectures had to be slightly modified to adapt them to this specific segmentation task, always using the validation set to assess the effectiveness of the changes. Our U2-Net did not require any modifications as it is already designed for photoreceptor segmentation in macular diseases^21^. Detailed descriptions of the architectures, the implementation and the training strategy are provided in Supplementary Material Section 2.

At test time, an unlabelled, full resolution raster scan (512 × 496) pixels was processed by each individual model, recovering four different score maps, one per model, in which a pseudo-probability of being part of the photoreceptors was assigned to each pixel coordinate. The pixel-wise average of these maps is taken in order to obtain a mean score map of the photoreceptors that represents the consensus among the different models. Similarly, the standard deviation is also computed pixel-wise to retrieve a disagreement map across all the automated evaluations. Finally, the average score map is thresholded using the Otsu method^22^ to retrieve a binary segmentation of the photoreceptors.

### En face thickness and standard deviation maps

The binary segmentations and the standard deviation maps are used to estimate en face thickness and model disagreement maps, respectively. The en face thickness maps provide an overall representation of the photoreceptors for the full OCT volume, which can be used to assess the variability in the photoreceptors density or to identify focal disruptions or other pathological changes. Similarly, the en face standard deviation map allows to easily identify areas of disagreement between models, which might be associated either with the presence of morphological pathology or with image artefacts. Moreover, using the en face representation, it is possible to briefly summarise the information of a full volume into a single image.

**Figure 3:**
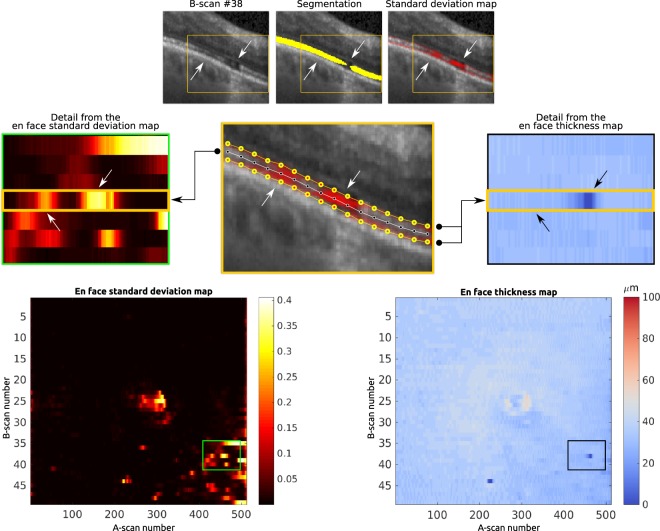
Schematic representation of the process for deriving the en face standard deviation (left) and thickness (right) maps from the B-scan level segmentations and standard deviations. The upper and lower interfaces of the photoreceptor layer (yellow dotted lines) are interpolated to retrieve two continues edges for the photoreceptors. A medial axis (white dotted line) is estimated based on the interfaces, and the standard deviation values on each pixel lying on this axis is used to produce the en face standard deviation map (left). The thickness is automatically computed for each A-scan by taking the distance between the two edges, while the thickness is set to 0 if there are disruptions (right).

  Figure 3 illustrates our approach for reconstructing both maps from the outputs obtained by the ensemble of the full set of B-scans from an OCT volume. In particular, given the outputs for a single B-scan, a B-spline is fit and positioned on the upper and lower interfaces of the segmented photoreceptors to retrieve a continuous representation of the layer, even under the presence of disruptions or holes in the segmentation. Then, the thickness of the layer is estimated by measuring the distance (in the y-axis) between both interfaces. Repeating this process for all the B-scans of an OCT volume achieves a full en face representation in which each row represents the thicknesses for each B-scan, and each column is the thickness for each A-scan.

For the standard deviation, most of the variation is expected to happen on the edges of the layer, which might be interesting for manual correction of the results at a B-scan level. However, we are interested in representing other variations in the pseudo-probabilities provided by each model, such as those occurring inside the photoreceptors area, which might be associated with high uncertainty due to disruptions or concurrent pathology. To this end, the central axis of the photoreceptors is estimated by taking the average position in between the upper and the lower interfaces of the layer. Then, the standard deviation values on these pixel coordinates are mapped to the corresponding position in the en face representation of the standard deviation map.

### Quantitative evaluation

The proposed approach was quantitatively evaluated in the full OCT volumes and in three different areas of the ETDRS grid: the central subfield (CSF), the 3 central millimetre (CMM) area and the 3–1 CMM ring. The pixel scores provided by the FCNNs were quantitatively evaluated using precision/recall curves^23^. Precision (Pr) is defined as the fraction of pixels that were correctly classified as belonging to the area of interest (e.g. the photoreceptors layer) (=true positives divided by the sum of true positives and false positives). Recall (Re, also known as sensitivity) accounts for the fraction of identified pixels of interest with respect to the total number of pixels belonging to the region (=true positives divided by the sum of true positives and false negatives). Pr/Re curves^23^ are similar to the receiver-operating characteristic (ROC) curves, in which a score map is thresholded at different levels and the resulting sensitivity and specificity values are depicted in a 2D plot. The key difference is that Pr/Re curves plot precision vs. recall values, instead, allowing a better analysis of classification or segmentation results where the class of interest is proportionally imbalanced with respect to all the other elements. In this case, the pixels belonging to the photoreceptors layer represent approximately 2% of the pixels of a B-scan, which results in a suboptimal usage of ROC curves. The area under the precision/recall curve (AUC) quantitatively summarises the performance of the method, with 1 associated to a perfect classification and 0 to a completely inverted result.

The binary segmentations obtained by thresholding the score maps were evaluated in terms of the Dice coefficient, Precision and Recall. Formal definitions of these metrics are provided in Supplementary Material Section 3. The Dice coefficient can be defined in terms of precision and recall^24^ as twice the product of precision and recall divided by their sum and is an appropriate overall indicator of the quality of the binary results. The mathematical formula including the definition of the Dice coefficient using the relationships between the segmentation and the annotation can be found in the Supplementary Material.

The results of the proposed approach were quantitatively compared with the performance of each of its constitutive models using the above-mentioned metrics. Such an assessment allows to study the contribution of model ensembling in improving the results. We studied the statistical significance of the improvements in the segmentation results using one-tail paired Wilcoxon sign-rank tests at a significance level of p < 0.05 (*n* = 12 volumes).

We also statistically assessed if the differences in the pixel-wise thickness estimation of the photoreceptor layer caused a bias in the prediction of the average thickness. To this end, we used a paired-sample *t*-test with a confidence level of 0.01 for each evaluation area of the ETDRS grid, and also on the full volume, comparing the average estimated thickness with the average thickness according to the manual annotations (*n* = 12 volumes). When the assumptions of the *t*-test were not held (e.g. data was not normally distributed according to an Anderson-Darling test or data was not homoscedastic according to a F-test, both at a significance level of 0.05), a paired Wilcoxon sign-rank test was used.

### Inter-observer variability

The inter-observer variability was assessed using the annotations produced by the second human expert in the sample of B-scans described before. These manual segmentations were compared with respect to the ground truth and evaluated using the Dice coefficient, Precision and Recall. By contrasting these results with those obtained by the proposed ensemble, we can study if the performance of the algorithm is in line with the one of a human expert, as evaluated using the same ground truth annotations and the same metrics. The statistical significance of the differences in performance was assessed using one-tail paired Wilcoxon sign-rank tests at a confidence level of 0.05 (*n* = 24 for individual samples, *n* = 72 when using the full sample).

## Results

### Quantitative evaluation

**Figure 4:**
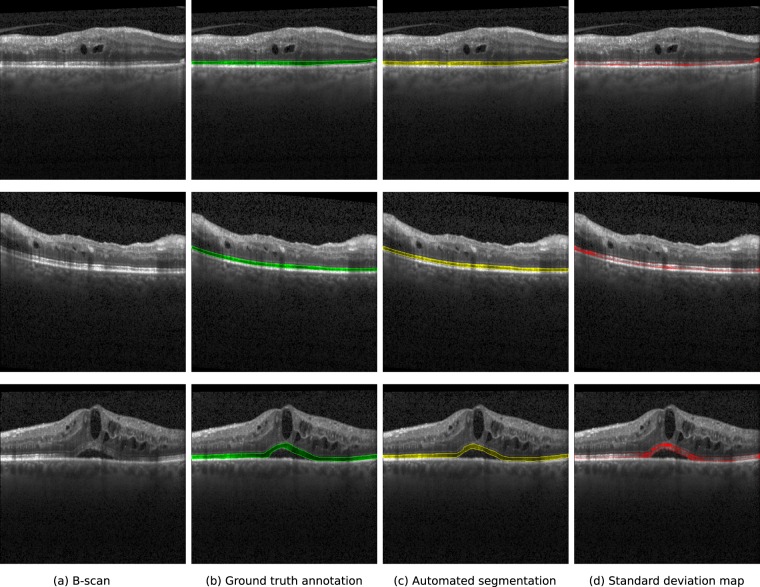
Qualitative examples of the outputs of our method in different B-scans. From left to right: original B-scan, ground truth annotation (green), segmentation (yellow) and standard deviation map (red) obtained using the ensemble. The areas in red in the last column correspond to the standard deviation values, normalised between 0 and 1 using their maximum and minimum values for better visualisation.

Examples of different B-scans, their associated ground truth annotations, segmentation results and standard deviations maps provided by our ensemble are depicted in Fig. 4. The segmentation approach performs similarly to the retina expert, even under the presence of cysts and subretinal fluid. The standard deviation maps exhibit high values of disagreement between models in areas with abnormal thinning (Fig. 4, top scan, right side of the B-scan), shadowing (Fig. 4, middle scan, left side of the B-scan) and in subtle interfaces due to pathologies (Fig. 4, bottom scan, centre of the B-scan). Notice also that the standard deviation map presents high values in the upper and lower boundaries of the photoreceptor layer, which is consistent with the ambiguities usually observed in manual annotation.

**Figure 5:**
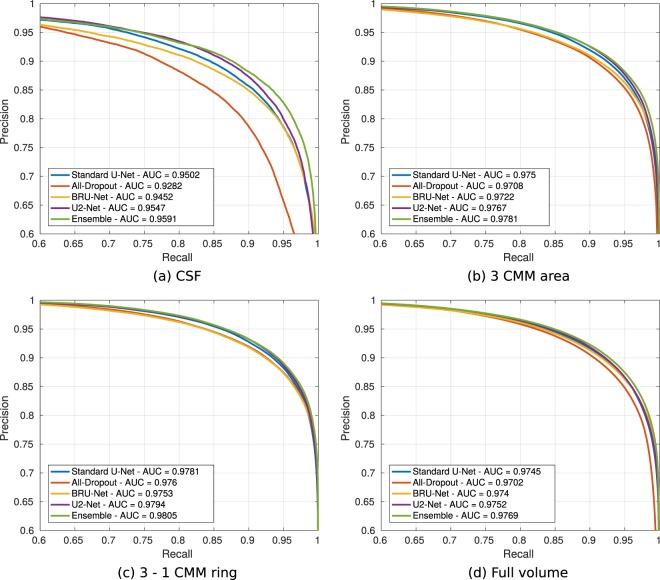
Precision/Recall curves and areas under the curves (AUC) obtained by each individual neural network and our ensemble, on each evaluation area from the ETDRS grid and on the full volume. The x and y axis correspond to the recall (sensitivity) and precision values as obtained by applying different thresholds over the score maps.

  Figure 5 depicts the precision/recall curves and their corresponding AUC values, as obtained by each CNN architecture and the ensemble on each area of the ETDRS grid, for the 12 volumes (588 B-scans) on the test set. A precision/recall curve dominates the others if it is closer to the right-up corner (precision = 1, recall = 1), which is also associated with an AUC value closer to 1. The ensemble reported the highest performance for all the evaluation areas, with the highest improvement evidenced in the CSF (AUC = 0.9591).

  Table 1 presents the average and standard deviation Dice, Precision and Recall values, as calculated on the test set from the binary segmentations. In all the evaluation areas, the ensemble reported the highest mean Dice coefficient compared to each separate CNN. This improvement was always statistically significant with respect to the performance of the All-Dropout network (p < 0.017). In the CSF, the ensemble reported the highest mean and median Dice values, a setting that is in line with the behaviour observed in Fig. 5. Such an improvement is significant when comparing the ensemble with the standard U-Net and the All-Dropout networks (p < 0.011). The differences in the Dice index with respect to our U2-Net are significant when evaluating on the full volume (p = 0.021). In the remaining areas of the ETDRS grid, the ensemble consistently outperformed its constitutive models (Dice coefficient). Compared to all the models and the ensemble, the BRU-Net reported the highest average recall or sensitivity, but the lowest average precision. On the other hand, the results of the All-Dropout network showed the lowest average recall with the highest average precision.

**Table 1:** Mean ± standard deviation values of Dice, precision and recall (sensitivity) for each individual model and our ensemble, as evaluated on the test set. Bolds indicate maximum values and asterisks indicate statistically significant differences with respect to the ensemble (p < 0.05) based on one-tail paired Wilcoxon sign-rank tests.

Metric	Evaluation area			
*Model*	CSF	3 CMM area	3–1 ring	Full volume
**Dice**				
*U-Net*	0.867 ± 0.075^*^	0.908 ± 0.028^*^	0.914 ± 0.026	0.908 ± 0.025^*^
*All-Dropout*	0.789 ± 0.175^*^	0.885 ± 0.049^*^	0.898 ± 0.032^*^	0.894 ± 0.036^*^
*BRU-Net*	0.865 ± 0.076	0.905 ± 0.027^*^	0.910 ± 0.029	0.908 ± 0.022^*^
*U2-Net*	0.874 ± 0.074	0.912 ± 0.026	0.917 ± 0.024	0.909 ± 0.024^*^
***Ensemble***	**0.875** ± **0.075**	**0.912** ± **0.026**	**0.917** ± **0.024**	**0.911** ± **0.024**
**Precision**				
*U-Net*	0.9160 ± 0.052	0.918 ± 0.043^*^	0.919 ± 0.044^*^	0.920 ± 0.039^*^
*All-Dropout*	**0.930** ± **0.053**	**0.936** ± **0.046**	**0.937** ± **0.048**	**0.939** ± **0.029**
*BRU-Net*	0.889 ± 0.050^*^	0.903 ± 0.050^*^	0.904 ± 0.053^*^	0.913 ± 0.036^*^
*U2-Net*	0.920 ± 0.047	0.923 ± 0.041	0.924 ± 0.043	0.925 ± 0.038
***Ensemble***	0.925 ± 0.041	0.923 ± 0.043	0.923 ± 0.046	0.926 ± 0.037
**Recall (Sensitivity)**				
*U-Net*	0.832 ± 0.116	0.900 ± 0.042	0.910 ± 0.034	0.898 ± 0.045
*All-Dropout*	0.708 ± 0.224^*^	0.842 ± 0.068^*^	0.864 ± 0.045^*^	0.856 ± 0.068^*^
*BRU-Net*	**0.854** ± **0.127**	**0.910** ± **0.035**	**0.918** ± **0.030**	**0.904** ± **0.042**
*U2-Net*	0.840 ± 0.117	0.902 ± 0.039	0.912 ± 0.033	0.895 ± 0.043
***Ensemble***	0.840 ± 0.122	0.904 ± 0.038	0.913 ± 0.030	0.899 ± 0.044

### Comparison with a second retina expert

**Figure 6:**
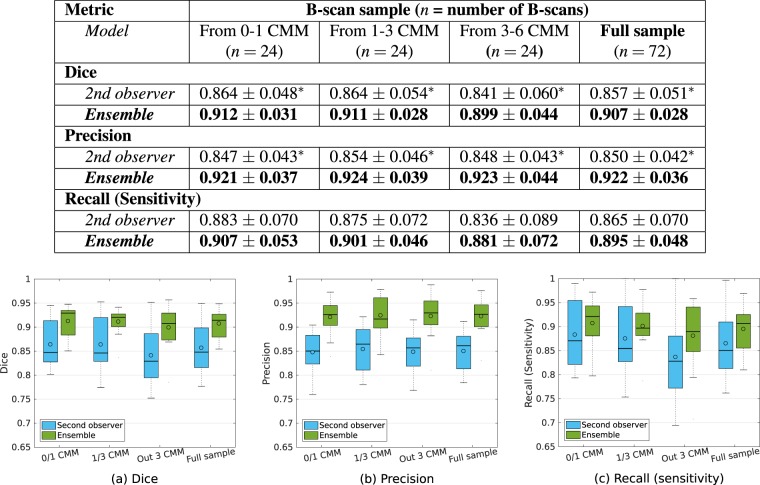
Top: Comparison of mean ± standard deviation Dice, precision and recall (sensitivity) values between the second retina expert and our ensemble, as evaluated on each random sample of B. n indicates the number of B-scans in the sample. Bolds are used to highlight the maximum mean values. Asterisks indicate statistically significant differences with respect to the performance of the ensemble (one-tail paired Wilcoxon sign-rank test, p < 0.05). Bottom: Boxplots comparing the results obtained by the second retina expert and the ensemble, as obtained on the random sample of B-scans. (**a**) Dice coefficient, (**b**) Precision, (**c**) Recall (sensitivity).

  Figure 6 presents the results obtained by the second human observer and the ensemble, as evaluated in terms of Dice, Precision and Recall (sensitivity) on each of the B-scans subsamples (0/1 CMM, 1/3 CMM and 3/6 MM) and the full sample. The ensemble consistently reported higher, average quantitative metrics in all the B-scan samples. Statistically significant differences were observed in the Dice coefficient (p < 0.022) and the Precision values (p < 0.003). The mean and median values of Recall (sensitivity) reported by the ensemble were higher than those obtained by the second expert. However, the differences were not statistically significant (p > 0.088).

### Thickness and standard deviation maps

The average thickness of the photoreceptors was computed for each area of the ETDRS grid based on the thickness maps calculated from the ground truth annotations and from the segmentations provided by our ensemble. Independently of the area of analysis, we observed that the differences in the average thickness values were not statistically significant (*t*-tests: p > 0.19 for the full volume, p > 0.26 for the 3 CMM area and p > 0.55 for 3–1 CMM ring; Wilcoxon sign-rank test: p > 0.01 for the CSF), which indicates that the errors of the automated approach did not affect the average thickness estimation.

**Figure 7:**
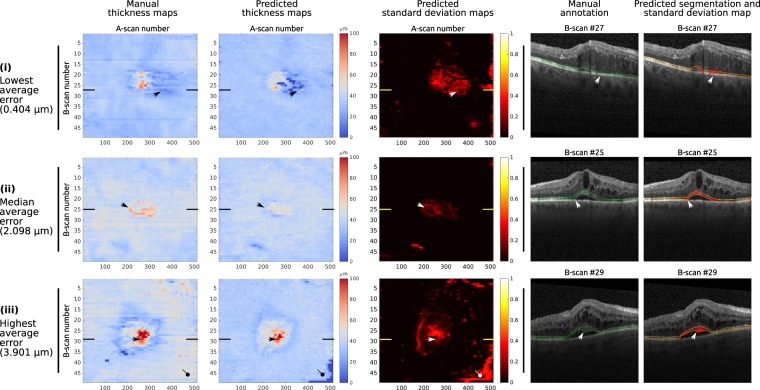
Clinical qualitative assessment of the en face thickness and standard deviation maps. From left to right: en face thickness maps derived from the manual annotations (in microns, *μm*); en face thickness maps derived from the binary segmentations obtained with the ensemble (in microns, *μm*); predicted en face standard deviation maps, as provided by the ensemble; representative B-scan extracted from the CSF of each corresponding volume, showing the manual annotation of the photoreceptors (green); same B-scan showing the binary segmentation (yellow) and the standard deviation map (red), as provided by the ensemble. From top to bottom: cases on the test set with the lowest (i), the median (ii) and the highest (iii) mean absolute pixel-wise error of the thickness in the full volume. The position of each B-scan is indicated by the bold lines in the en face thickness and standard deviation maps. The OCT scan (i) shows extensive intrarretinal fluid (IRF), central subretinal fluid (SRF) and hyperreflective exudation in a patient with DME. Photoreceptor layer thickening occurs above SRF with thinning at its margins. Deviations between predicted and manually annotated photoreceptor thickness occur in regions where exact delineation of photoreceptor layer borders shows to be extremely difficult due to profound damage of photoreceptors as well as overlying retinal structures. A similar behaviour is observed in the OCT scan presented in (ii), which corresponds also to a patient with central IRF and SRF, this time due to RVO. Finally, the OCT scan (iii) is affected by IRF and SRF with concomitant alteration of the photoreceptors layers due to central RVO. Central photoreceptor layer thickening–partly underestimated by the ensemble (black and white arrows) but captured by the standard deviation map as an abnormality–occurs above SRF and photoreceptor layer thinning at its margins. The predicted maps show the ability of the model to capture this overall pattern of photoreceptor thickness variation in a seemingly smoothing fashion. The differences in thickness at the edges (also observed in the standard deviation map) correspond to shadowing of the photoreceptor layer due to a large retinal vessel (arrow with circle tip).

In order to clinically assess the information provided by the en face thickness and standard deviation map, three representative volumes were qualitatively analysed in Fig. 7.

**Figure 8:**
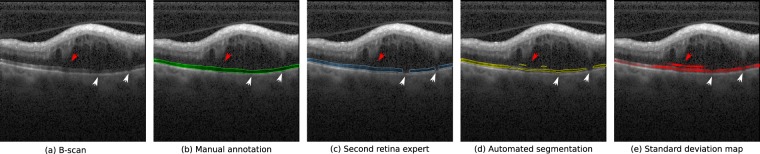
Failed segmentation. From left to right: (**a**) original B-scan, (**b**) manual annotation (green), (**c**) second human expert annotation (blue), (**d**) automated segmentation obtained by the ensemble (yellow), and (**e**) predicted photoreceptor interfaces from the segmentation (yellow) and standard deviation map (red). White arrows indicate areas where both retinal experts disagreed. Red arrow indicates small false positive detections of the photoreceptor layer.

Finally, Fig. 8 depicts a representative example of a B-scan with ambiguous results in which the algorithm performs poorly. The B-scan corresponds to a patient in the test set affected with branch RVO. Marked differences in manual and automated photoreceptor layer thickness can be noted in the upper quarter of the scan. In the area beneath intraretinal cystoid fluid the algorithm seems to fail, resulting in underestimated photoreceptor layer thickness. Presumably, shadowing by cystoid spaces decreases the segmentation performance, which also becomes evident in marked differences between the two observers concerning layer disruptions (red arrows). Moreover, the algorithm detected small false positive segments in the cystoid area (white arrows). The standard deviation map captures both areas of ambiguity and therefore serves as an indicator of where manual inspection might be needed.

## Discussion

The retinal photoreceptor layer is the primary anatomical site where incident light is converted into perception and vision. The photoreceptor layer consists of characteristic hyper- and hyporeflective bands, namely the inner segments/outer segments line–also known as the ellipsoid zone–, the interdigitation zone and the photoreceptor end tips. In total, this region comprises a morphological layer with a thickness of about 30–40 *μm*. In earlier years, the resolution of OCT devices was not sufficient to distinctly visualise the photoreceptor. Thus, focal disruptions or thickness variations were not identifiable for feature analysis. When spectral-domain OCT was introduced, visualisation of the different photoreceptor bands became possible, and controversial discussion regarding appropriate anatomical labelling has started, which is still ongoing^11^. Regardless of how these layers are called, there is a clear consensus that the integrity of the photoreceptor bands has a substantial impact on vision. However, it is unclear to which extent photoreceptors can be restored after morphological disruption, and which degree of disruption or thickness variability is functionally relevant and leads to permanent visual compromise^25–28^. Particularly in DME, the condition of the outer retinal layers, containing the photoreceptors, has been shown to better correlate with vision than central retinal thickness^29^.

A qualitative visualisation of the photoreceptor layer by OCT imaging alone does not offer solid insight in daily clinical routine. The mere segmentation of a healthy photoreceptor layer is already time-consuming and therefore unfeasible during routine examinations. Furthermore, if the photoreceptor layer shows alterations, its segmentation and quantification is significantly prone to errors. As a consequence, the inter-expert variability is higher in the challenging task of pathologic biomarker annotation than for labelling healthy retinal structures^30^. Most publications correlating photoreceptor structures with vision have used single a-scan annotations to measure layer thickness or have only used a subjective grading by a clinician to judge on the photoreceptor integrity, which does not offer a reliable or quantitative structure/function correlation.

In this study, we have introduced an automated deep learning method for segmenting and quantifying the photoreceptors in OCT scans of patients with DME and RVO, diseases with significant disturbance of intraretinal morphology. Only an automated annotation of photoreceptor integrity and its thickness in particular, can reliably identify the level of photoreceptor alteration in its current status and during follow-up. Few attempts were made in the past, yet all of them differ from ours in several key aspects. Chan *et al*.^31^ presented the first approach for photoreceptor segmentation in healthy scans, based on standard statistical learning algorithms (e.g. logistic regression) and manually engineered features. Alternatively, we have presented a deep learning approach that is able to properly identify the area of interest not only in healthy regions, but also in areas with variable alterations due to the underlying disease such as RVO or DME. The only available deep learning method which is successful in pathological OCT scans was our own U2-Net model, recently published^21^.

Our automated approach is built on the concept of multi-grader averaging. To identify the photoreceptor layer manually, certified experts annotate the regions of interest. Then, the average of these annotations can be taken as a more realistic representation of the photoreceptor layer condition. Typical OCT artefacts such as noise, saccadic motions and vessel shadows might be misinterpreted by human readers as layer disruptions, while certain bands such as the interdigitation zone (IZ) might not be easily separated from the RPE, leading to inter-expert variability. Furthermore, as previously mentioned, abnormal cases with multiple concomitant lesions such as intra- and subretinal fluid are much more difficult to interpret and annotate than healthy scans. The proposed algorithm is able to alleviate this issue. Our human-inspired hypothesis ensembling the outputs of different fully convolutional, U-shaped neural network architectures allows to achieve consistently better results than using each model individually and has been confirmed during extensive evaluation on 12 comprehensive OCT volumes including a total of 588 B-scans. Our approach provides not only an accurate morphological segmentation of the photoreceptor band, but also gives qualitative feedback about the disagreement between its constitutive models, allowing experts to realistically identify potential areas of errors. Furthermore, fully-automated approaches usually have a much higher intra-grader reproducibility, whereas the manual task of segmenting retinal structures does not offer high reproducibility values^32^. This is of major importance when clinical decisions have to be made during early diagnosis and long-term follow-up and an individual prognosis of disease severity and/or therapeutic outcomes is needed.

The data applied both for training and evaluating the proposed approach correspond to OCT volumes collected from several clinical studies, meaning that their quality is overall in line with the one expected from clinical routine examinations. nevertheless, anatomical and disease related artifacts were rather common in this highly pathological data. In particular, Fig. 4 depicts exemplary B-scans with areas of poor EZ signal due to vessel and cyst shadows. Our ensemble model exhibited good performance on these scenarios, particularly when the discontinuities in the signal are not long (Fig. 4, second and third rows). On the contrary, the model was observed to undersegment the photoreceptor layer under large vessel shadows (Fig. 7(iii)) or in scans with strong signal attenuation due to fluid accumulation (Fig. 8). Nevertheless, it is worth mentioning that the standard deviation maps were still able to effectively recognize those areas, indicating the need of potential manual correction.

A substantial inter-observer variability was observed in manual photoreceptor annotation, particularly in scans with extensive macular edema like the one shown in Fig. 1. This motivated the comparison of the algorithm performance to a second retina expert, to put the results within the context of inter-observer variability. We quantitatively observed that our proposed approach achieves superior Dice values, specially due to higher precision, compared to the annotations produced by a second retina expert that individually labelled the images. Given these results, we envision that this automated segmentation tool might be primarily of clinical use in evaluating the state of the retinal photoreceptors after the initial treatment phase, i.e., when majority of the baseline retinal fluid is cleared and the expert agreement is higher, to evaluate the prospect of recovering the vision under subsequent longer-term treatment.

The interpretation of our method is also much better explainable to clinicians than a stand-alone black-box segmentation output of a single model. We have proposed to automatically compute model disagreement maps (standard deviation between the score maps provided by each constitutive model of the ensemble) that can be used to carefully analyse specific B-scans. Highlighting areas of disagreement between the automated observers in an en-face fashion indicates potential errors in their individual segmentations. We also observed that most of the variabilities are explained by the true presence of pathologies (e.g. disruptions or thickening of the photoreceptors). Therefore, it is more likely that the disagreement maps highlight areas where disease activity or photoreceptor loss has occurred and is responsible for vision loss. Regions where all segmentations are the same and non-questionable correspond more reliably to healthy areas of the photoreceptor layer, as we observed in our experiments. In few cases, the disagreement occurred due to image artefacts such as vessel shadowing, which is easily recognised by the clinician when the maps are presented together with an en-face retinal image containing the vessel structures (e.g. the fundus image taken together with each OCT volume).

We have also presented a strategy for automatically computing en-face thickness maps of the photoreceptors. This approach is also applicable to study the effect of pathological changes in photoreceptor thickness due to other diseases such as glaucoma^33^ or genetic dystrophies such as retinitis pigmentosa^25,34^, or for estimating other morphological properties such as the overall length of photoreceptors associated with several diseases, including DME^29^ and RVO^28^ or even early age-related macular degeneration. Our automated photoreceptor segmentation could be prospectively applied in clinical studies and allows for the first time to segment the photoreceptors on each individual voxel of the raster scan instead of using random point thickness measurement obtained manually. The method can also allow the interpretation of retrospective studies in which pathognomonic changes in photoreceptor morphology are assessed during pharmacological treatments^12^.

Leveraging the outputs of different models reproduces the idea of conclusively integrating the judgement of multiple experts, although it is more efficient and less expensive. Despite the fact that this principle is not novel scientifically, not many studies have taken advantage of this in the past, and none of them for photoreceptor segmentation. In particular, Kamnitsas *et al*. used an ensemble of neural networks for brain tumour segmentation that reached the first place in the MICCAI-BRATS contest^35^. Similar ideas were recently explored in other OCT imaging-based tasks such as identifying geographic atrophy lesions^36^ or segmenting artefacts^37^.

The proposed approach is sufficiently general to incorporate any deep learning model for segmentation. The decision for the proposed networks was based on its popularity at the time of use and the judgement of the authors to obtain useful results for this segmentation task. The selected networks seem to be appropriate, according to the results. The All-Dropout network achieved high precision but low recall, meaning that it is unable to retrieve some pathological photoreceptors. The opposite behaviour was observed in the BRU-Net, which produced segmentations with a larger number of false positives. The classical U-Net performed similarly in the different evaluation areas, although with higher precision. Compared to all others, the U2-Net achieved better results, which is consistent with the fact that it was especially designed for photoreceptor segmentation in RVO, DME and AMD. In this paper we build on top of our previous contribution (U2-Net) by integrating the outcomes of other artificial intelligence approaches that were specifically adapted to the challenging task of photoreceptor segmentation. By means of this approach, we were able to achieve better results and exploit the individual advantages of each network, taking automated feature segmentation at the level of the retina a large step further.

In conclusion, we have introduced a deep learning-based convolutional neural network ensemble for automatically segmenting and quantifying the photoreceptors in routine OCT images of patients with DME and RVO. This achievement is most relevant in research and clinical routine as it offers a reliable interpretation of the photoreceptor condition for each individual and at each time. Identification of photoreceptor alteration may strongly improve our insight into the pathophysiology of retinal/macular disease and allow a quantification of functional loss based on true morphology.

## Supplementary information

Supplementary information

## Data Availability

The data sets used to train and evaluate our method cannot be shared at the current time due to data confidenciality agreements and sharing restrictions from data sources.

## Code availability

Code with the implementation of the neural networks used in the ensemble is publicly available at https://github.com/ignaciorlando/photoreceptor-segmentation.git.
