# Supplementary material for: Automated Quantification of Photoreceptor alteration in macular disease using Optical Coherence Tomography and Deep Learning
Source: Sci Rep. 2020 Mar 27;10:5619. doi: 10.1038/s41598-020-62329-9 (PMC7101374; doi:10.1038/s41598-020-62329-9)
Supplement: Supplementary file 1 — Supplementary information [file 41598_2020_62329_MOESM1_ESM.pdf]

Automated Quantification of Photoreceptor alteration in macular disease using Optical Coherence Tomography and Deep Learning

José Ignacio Orlando [a,#], Bianca S. Gerendas [a,#], Sophie Riedl [a], Christoph Grechenig [a], Anna Breger [b], Martin Ehler [b], Sebastian Waldstein [a], Hrvoje Bogunović [a] and Ursula Schmidt-Erfurth*

[a] *Department of Ophthalmology, Medical University of Vienna, Vienna, Austria*
[b] *Department of Mathematics, University of Vienna, Vienna, Austria*

# Equal contribution
** Corresponding author*

---

1. Formal definition of an ensemble

Formally, let $\mathbf{x} \in \mathbb{R}^{n \times m}$ be a B-scan of an OCT scan with n A-scans of length m, and $\mathbf{y} \in \{0,1\}^{n \times m}$ its associated manual annotation, where 0 represents the background and 1 the photoreceptor layer. Let also $\phi(\mathbf{x}; \theta)$: $\mathbb{R}^{n \times m} \rightarrow \{0,1\}^{n \times m}$ be a U-shaped fully convolutional neural network (FCNN) that maps the input image to a probability map, based on a series of parameters θ. These parameters are learned from a training set $S = \{(\mathbf{x}^{(i)}, \mathbf{y}^{(i)})\}, 1 \leq i \leq N$ by finding the configuration that minimizes a loss function:

$$J(\phi(\mathbf{x}; \theta), \mathbf{y}) = \frac{1}{N} \sum_{i=1}^{N} \mathcal{D}(\phi(\mathbf{x}^{(i)}; \theta), \mathbf{y}^{(i)})$$

where $\mathcal{D}(\cdot)$ stands for the the cross-entropy loss [1], which measures the difference between the predicted score map and its corresponding ground truth annotation. Due to the differences in the design of each network, it is expected that each $\phi_k(\mathbf{x}; \theta_k)$ provides a slightly different score map for a given image $\mathbf{x}$. To retrieve the most probable map, an ensemble $\bar{\phi}(\mathbf{x})$ can be defined by averaging the outputs of the K different models:

$$\bar{\phi}(\mathbf{x}) = \frac{1}{K} \sum_{k=1}^{K} \phi_k(\mathbf{x}; \theta_k).$$

This mean score map $\bar{\phi}(\mathbf{x})$ is then thresholded using the Otsu method [2] to retrieve a binary segmentation of the photoreceptors.

A map of the disagreement between the different networks, denoted by $\bar{\phi}_\sigma(\mathbf{x})$, could be obtained by taking the standard deviation of the outputs of each model, at a pixel level:

$$\bar{\phi}_\sigma(\mathbf{x}) = \sqrt{\frac{\sum_{k=1}^{K}\left(\phi_k(\mathbf{x};\theta_k) - \bar{\phi}(\mathbf{x})\right)^2}{K-1}}.$$

Thus, the areas where the differences between the score maps are negligible will present low values in $\bar{\phi}_\sigma(\mathbf{x})$, whereas the regions with high variability between models will result in higher deviation values. This outcome can be interpreted as a pixel-wise representation of the disagreement or uncertainty of the ensemble, which might be applied to guide the manual assessment of the results.

2. Implementation details of the models used in the ensemble

In this section we discuss the intuition behind our segmentation method based on an ensemble of U-shaped fully convolutional neural networks.

U-shaped FCNNs [3] are architectures that are characterized by using only convolutional and pooling layers, and by presenting two well defined branches, namely the encoder and the decoder (Figure 1). The encoder is a contractive path that gradually reduces the size of the image and quantitatively characterize the relevant features of the region of interest. On the other hand, the *decoder* is an expansive path that takes the outputs of the encoder as an input, and reconstruct the segmentation at the same image resolution as the input. The encoder and the decoder are connected by the bottleneck layer, which is located in the output of the last layer of the encoder path, and by a series of *skip connections* that allows the decoder to integrate and analyze features from higher resolutions.

The U-Net [3] was the first architecture that introduced the U-shaped structure with skip connections. One important limitation of this network is its restrictive *receptive field*, which corresponds to the overall area that can be analyzed by the encoder to identify the relevant features of the region of interest. This means that is difficult for the classical U-Net to learn non-local properties from the structure, but only small-scale characteristics of the structure to segment, instead. In [4], the authors proposed to overcome this issue by introducing the Branch-Residual U-Net, or BRU-Net, a modified U-Net for segmenting the retinal layers in pathological OCT scans. In particular, the so-called dilated convolutions [5] were introduced to this architecture in order to increase its original receptive field. This setting improved its ability to deal with lesions such as subretinal or intrarretinal fluid. In addition to this novel feature, the authors introduced branch residual connections [6] and batch normalization [7] to allow faster convergence during training.

Another key aspect of deep neural networks is their *generalization ability*, since our purpose is to learn models sufficiently general to be applied on different databases. This setting is usually enforced during training by applying *regularization techniques* that are intended to avoid the CNNs to learn too specific details but more general features, instead. The original U-Net was regularized by only applying dropout [8] in the bottleneck layer of the network. Alternatively, Novikov *et al.* [1] have recently presented an alternative architecture, namely

All-Dropout, that uses dropout after each convolutional layer. This approach demonstrated to be more accurate for segmenting anatomical structures in chest X-ray scans due to the major improvement in its generalization ability thanks to such a heavy regularization.

Finally, our fourth U-shaped architecture is our U2-Net [9]. This network was introduced for photoreceptor segmentation in OCT scans of patients with DME, RVO and AMD. It is inspired by the original U-Net, but includes leaky ReLU activation units [10] and dropout after some of the encoder/decoder layers. Moreover, it replaces the transposed convolutions in the decoder by upsamplings followed by a convolutional layer. Instead of using n Monte Carlo samples as in [9], we only used a single one.

The inputs of these networks are full resolution B-scans of size 512 x 496 pixels, and the outputs are pseudo-probability or score maps of the photoreceptors, with the same size as the input. The full pipeline was developed using Python (Python Software Foundation, https://www.python.org/) as programming language, and implemented using the deep learning framework Pytorch 0.4.0 (https://pytorch.org/). All the models were trained using an NVIDIA Titan X GPU (Nvidia, CA, USA). We slightly modified some of the architectures for this specific application. The implementation details of each network are provided in the sequel.

2.1. U-Net implementation

Figure 2 depicts a graphical representation of our U-Net architecture [3]. The encoder path is composed of 5 convolutional blocks made of two consecutive convolutional layers with 3 x 3 filters, with batch normalization and ReLUs as activation units. After each of the first 4 convolutional blocks, a max-pooling layer reduces the resolution of its input to half of its original size. The decoder path consists in 4 deconvolutional blocks. Each of this blocks consists in three operations: a nearest neighbor upsampling with a factor of 2, a concatenation between the upsampled input and the output of the skip connection, and a 3 x 3 convolutional block without batch normalization. The last convolutional block is followed by a convolutional layer with filter of size 1 x 1, and its outputs are transformed using a softmax operation to obtain the score maps for the background and the photoreceptors classes. Dropout with a probability of 0.5 was used only in the bottleneck layer.

The main changes compared with the original U-Net are the usage of (i) nearest neighbor upsampling followed by two convolutional layers, and (ii) batch normalization after each convolutional layer. Both changes were proposed in [9] to improve its original performance.

2.2. All-Dropout implementation

The All-Dropout architecture [1] used as part of our ensemble is depicted in Figure 3. Its general organization is similar to the original U-Net, as it also consists in 5 convolutional blocks followed by 4 upsampling blocks, with skip connections. However, notice that dropout with a probability of 0.2 is applied after each convolutional block. This setting imposes heavy regularization during training, which forces the network to learn sufficiently general filters

that are more accurate for detecting the photoreceptors in healthy scenarios in which no disruptions or neighboring lesions are observed.

2.3. BRU-Net implementation

The third architecture integrated in our ensemble is inspired by the BRU-Net [4] (Figure 4). Despite the fact that the overall organization of the network is still U-shaped, it introduces several modifications with respect to the original U-Net. The input image is processed by an input block (Figure 4, middle-left) consisting on a convolutional layer with 5 x 5 filters, followed by batch normalization and a ReLU activation unit. The output responses are the inputs for the encoder path, which is based on the continuous application of 5 block-d blocks (Figure 4, bottom-left). Each block-d consists in a 1 x 1 convolutional layer with N output filters, that is further processed in parallel by 3 different dilated convolutional layers with filters of the same size (3 x 3 pixels) but with different dilation factors (1 x 1, 3 x 3 and 5 x 5). These layers are applied in parallel, and the outputs of all these operations and the initial convolution are summed up and go through batch normalization and a ReLU activation unit. The resulting map is then processed by an additional convolutional layer with N filters of size 1 x 1, and a max-pooling operation with filters of size 2 x 2 pixels is finally applied to reduce its resolution by a factor of 2. The outputs of each block-d are always concatenated with a downsampled version of the input image obtained by applying average pooling at different resolutions. These operations are repeated until reaching a resolution of 16 x 16 pixels. From there, a series of 5 *block-u* operations are applied. The structure of these blocks is the same as the one used for the block-d, although a nearest neighbor upsampling operation is applied to the outputs of the 1 x 1 convolutions instead of a max-pooling. This allows to restore the resolution, which is necessary to concatenate these outputs with the feature maps provided by the skip connections. Only in the first block-u, dropout with a rate of 0.5 is used before upsampling. It is worth mentioning that instead of propagating only the outputs of the block-d's, the skip connections propagate the concatenation between these maps and the original image. This is repeated until restoring the original image resolution. Finally, an output block (Figure 4, bottom-right) is applied to retrieve the score maps for the photoreceptors. This block consists in a convolutional layer with 3 x 3 convolutions, followed by batch normalization, a ReLU activation unit and a 1 x 1 convolutional layer. Notice that instead of generating a 1-D output as in the original proposal [4], our BRU-Net outputs a 2D score map with the scores for the photoreceptors and the background. We followed such an approach because we used the cross-entropy loss instead of the mean squared error loss. We also used an exponential increasing number of filters instead of the Fibonacci sequence used by the original architecture. This setting allows the networks to learn a larger number of parameters. The bottleneck layer used 512 instead of 1024 filters due to GPU memory restrictions

2.4. U2-Net implementation

We used exactly the same implementation as in [9] (Figure 5). However, instead of taking N Monte Carlo samples as in [9], we restrict ourselves to produce a single output. Our experiments using more samples resulted in less accurate results of the ensemble when evaluating it on the validation set.

2.5. Training configuration

Our four networks were trained using the cross-entropy loss as objective function, with a batch size of 2 B-scans. The loss was minimized using Adam optimization [11], with an initial learning rate of $\eta = 10^{-4}$. Weight decay ($\lambda = 5\times10^{-5}$) and data augmentation (horizontal flippings) were applied to reduce overfitting. The validation loss was used to assess the overall performance during training but also to further guide the learning process. In particular, the learning rate was decreased by a factor of 2 up to a minimum of 10^{-7} if the validation loss was not improved in the last 10 epochs. The best model out of 160 epochs was taken as the final model.

3. Formal definition of quantitative metrics

In this section we formally defined the quantitative evaluation metrics for segmentation.

Precision is defined as the ratio between true positives (TP) and the sum of true positives (TP) and false positives (FP):

$$\mathrm{Pr} = \frac{TP}{TP + FP}$$

Recall (also known as sensitivity, Se) is defined as the ratio between the true positives (TP) and the sum of true positives (TP) and false negatives (FN):

$$\mathrm{Re} = \mathrm{Se} = \frac{TP}{TP + FN}$$

Dice index (also known as F1-score) can be defined as the harmonic mean between precision (Pr) and recall (Re). It can also be defined as two times the amount of pixels in the intersection between the segmentation (S) and the reference annotation (R) divided by the sum of pixels of the segmentation (S) and the reference annotation (R):

$$\mathrm{Dice} = F1 = 2 \times \frac{\mathrm{Pr} \times \mathrm{Re}}{\mathrm{Pr} + \mathrm{Re}} = \frac{2|S \cap R|}{|S| + |R|}$$

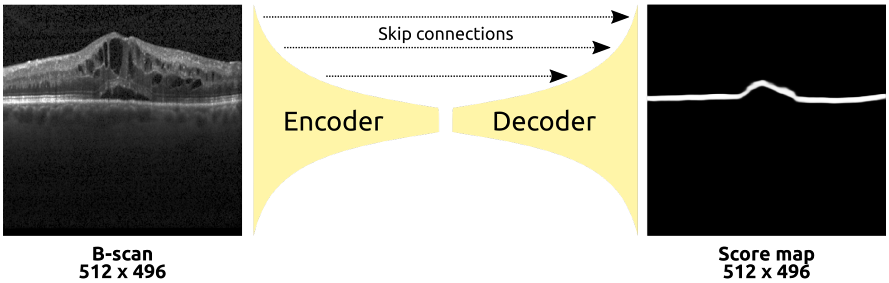

Figure 1: Schematic representation of a U-shaped fully convolutional neural network. These networks are composed of an encoder and a decoder path with skip connections. In our setting, the input is a Spectralis B-Scan (512 x 496 pixels), and the output is a pixel-wise score map of the photoreceptor layer.

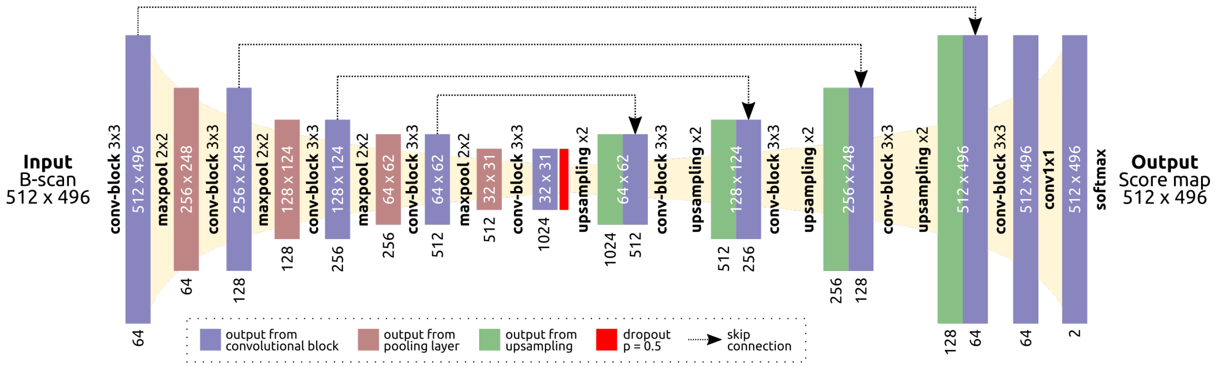

Figure 2: Our U-Net architecture.

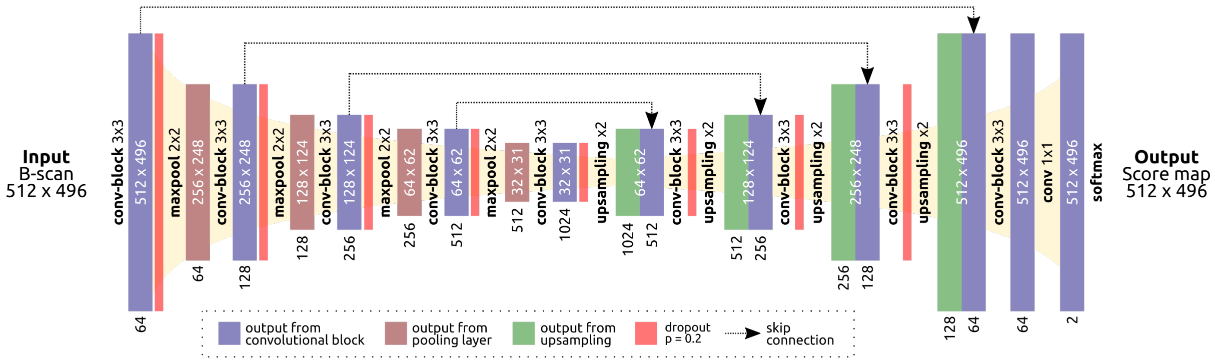

Figure 3: Our implementation of the All-Dropout architecture.

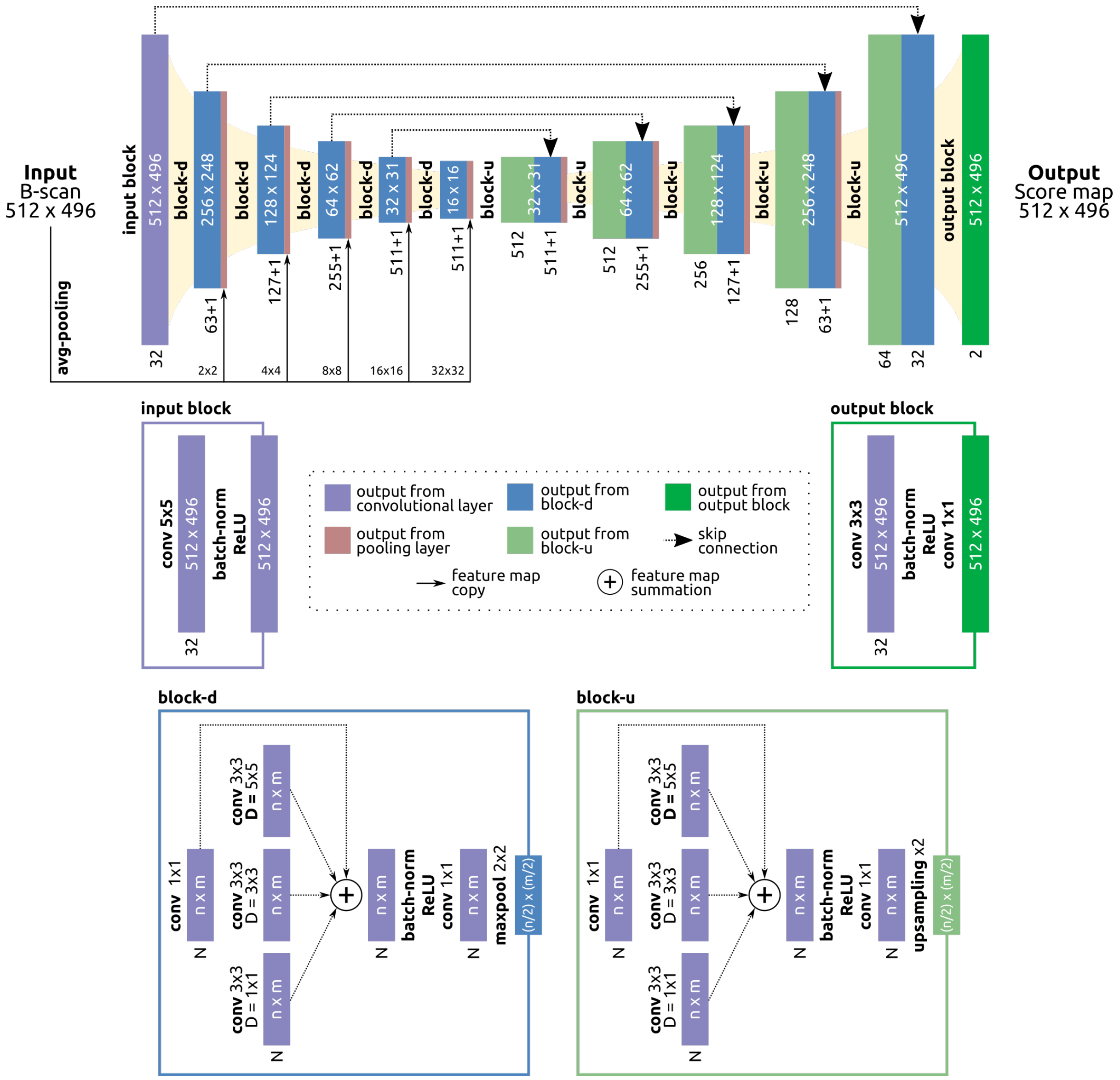

Figure 4: Our implementation of the All-Dropout architecture. Dropout with a rate of 0.5 is used in the first block-u, after upsampling.

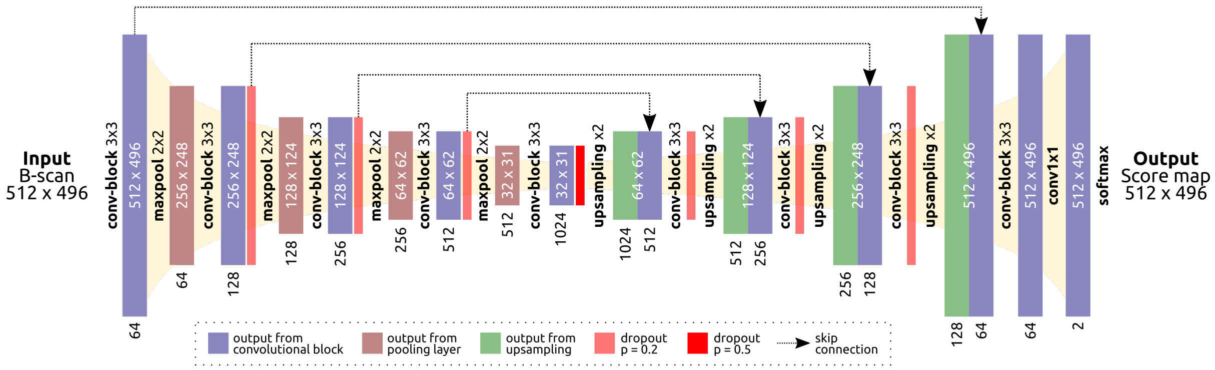

Figure 5: U2-Net architecture.
